# Phytic Acid Delays the Senescence of *Rosa roxburghii* Fruit by Regulating Antioxidant Capacity and the Ascorbate–Glutathione Cycle

**DOI:** 10.3390/ijms26010098

**Published:** 2024-12-26

**Authors:** Boyu Dong, Yulong Chen, Chengyue Kuang, Fangfang Da, Xiaochun Ding

**Affiliations:** 1 School of Chinese Ethnic Medicine, Guizhou Minzu University, Guiyang 550025, China; 15772710928@163.com (Y.C.); kcy20000128@163.com (C.K.); dafangfang@gzmu.edu.cn (F.D.); 2 Key Laboratory of Guizhou Ethnic Medicine Resource Development and Utilization, Guizhou Minzu University, State Ethnic Affairs Commission, Guiyang 550025, China; 3 Engineering Research Center for Fruit Crops of Guizhou Province, Engineering Technology Research Centre for Rosa Roxburghii of National Forestry and Grassland Adminstratio, College of Agriculture, Guizhou University, Guiyang 550025, China

**Keywords:** *Rosa roxburghii*, phytic acid, antioxidant capacity, ascorbate–glutathione cycle

## Abstract

*Rosa roxburghii* fruit has a short postharvest shelf life, with rapid declines in quality and antioxidant capacity. This research assessed how phytic acid affects the antioxidant capacity and quality of *R. roxburghii* fruit while in the postharvest storage period and reveals its potential mechanism of action. The findings suggested that phytic acid treatment inhibits the production of malondialdehyde (MDA) and enhances the activities and expressions of glutathione peroxidase (GPX), peroxidase (POD), catalase (CAT), and superoxide dismutase (SOD) while decreasing the generation of superoxide anions (O_2_^•−^) and hydrogen peroxide (H_2_O_2_). Phytic acid treatment activates the ascorbate–glutathione (AsA-GSH) cycle and enhances the activity and expression of key enzymes in the cycle: ascorbate peroxidase (APX), glutathione reductase (GR), dehydroascorbate reductase (DHAR), and monodehydroascorbate reductase (MDHAR). It also increases the levels of non-enzymatic antioxidants, such as ascorbic acid (AsA) and glutathione (GSH), while reducing the production of dehydroascorbic acid (DHA) and oxidized glutathione (GSSG). Moreover, phytic acid treatment enhances the ratios of AsA/DHA and GSH/GSSG, maintaining the reduced state of the fruit. In summary, phytic acid improves antioxidant defense system and activates the AsA-GSH cycle, alleviating oxidative damage and ensuring *R. roxburghii* fruit quality after harvest.

## 1. Introduction

*Rosa roxburghii*, primarily distributed in the southwestern regions of China, is a medicinal and edible plant with highly nutritious fruits [1]. The fruits contain a variety of bioactive compounds, including vitamin C, free amino acids, polysaccharides, phenolic substances, and more. In addition, *R. roxburghii* exhibits strong antioxidant activity, with an ascorbic acid content as high as 2000 mg/100 g, making it one of the highest sources of ascorbic acid [2,3]. The harvest period for fresh *R. roxburghii* fruits is short, typically occurring between August and October each year. Due to the lack of effective postharvest storage methods, the fruits quickly undergo ripening and aging, leading to negative changes in their quality, including the rotting and loss of nutritional content, resulting in significant economic losses [4]. In order to minimize postharvest losses of *R. roxburghii* fruits, it is imperative to investigate methods for halting the deterioration of fruit quality.

Reactive oxygen species (ROS) have both beneficial and detrimental effects on plant growth and development [5]. In plants, hydrogen peroxide (H_2_O_2_) at low concentrations functions as a signaling molecule that activates defense responses and is crucial for controlling growth, development, and stress tolerance [6]. Excessive ROS concentrations can trigger lipid peroxidation, protein unfolding, and DNA damage, which may result in the death of cells [7,8]. An antioxidant defense mechanism has developed in plants to control the ratio of ROS generation to detoxification. Superoxide dismutase (SOD), peroxidase (POD), catalase (CAT), and glutathione peroxidase (GPX) are important ROS-scavenging enzymes that cooperate to eradicate ROS and preserve homeostasis [9,10]. Additionally, the ascorbate–glutathione (AsA-GSH) cycle is an essential part of the antioxidant network in plants [11]. This cycle involves antioxidant compounds such as ascorbic acid (AsA) and glutathione (GSH), along with key enzymes like ascorbate peroxidase (APX), glutathione reductase (GR), dehydroascorbate reductase (DHAR), and monodehydroascorbate reductase (MDHAR). Previous studies confirmed that the activation of the AsA-GSH cycle can effectively enhance the antioxidant capacity of various fruits and vegetables, such as bell pepper [12], fresh-cut Chinese water chestnuts [13], pears [14], and fresh-cut pitaya [15], helping to maintain their quality.

Phytic acid is a highly phosphorylated molecule that is widely distributed in plants; it has been applied in food preservation and storage and is generally recognized as safe, according to previous reports [16]. Phytic acid plays an effective role in scavenging free radicals and advancing fruit quality [17,18]. By regulating ROS levels, phytic acid helps decrease the incidence of sour rot in table grapes [5]. Additionally, the mixture of phytic acid and 1-methylcyclopropene can inhibit browning in fresh-cut peaches, helping to maintain quality [19]. However, there were no reports on the application of phytic acid in the storage of *R. roxburghii* after harvest, and its preservation mechanism remains unclear.

The aim of this study was to address the challenge of the postharvest quality loss and shelf-life limitation of *R. roxburghii*, a fruit with significant nutritional and commercial value. By exploring the potential of phytic acid as a treatment, this study investigated its effects on ROS scavenging, the AsA-GSH cycle, and the overall quality of *R. roxburghii* fruits stored at room temperature. We hypothesized that phytic acid can enhance the antioxidant defense system, reduce oxidative stress, delay quality deterioration, and ultimately extend the shelf life of the fruit. This research provided new insights into the physiological processes involved in postharvest preservation, offering a promising strategy to reduce losses and improve fruit storage. The findings of this study contributed to the development of effective, natural preservation methods for *R. roxburghii*, which could benefit both the agricultural industry and consumers by extending storage periods, maintaining fruit quality, and improving marketability while reducing waste.

## 2. Results

### 2.1. Influence of Malondialdehyde (MDA) Content in R. roxburghii

**Figure 1:**
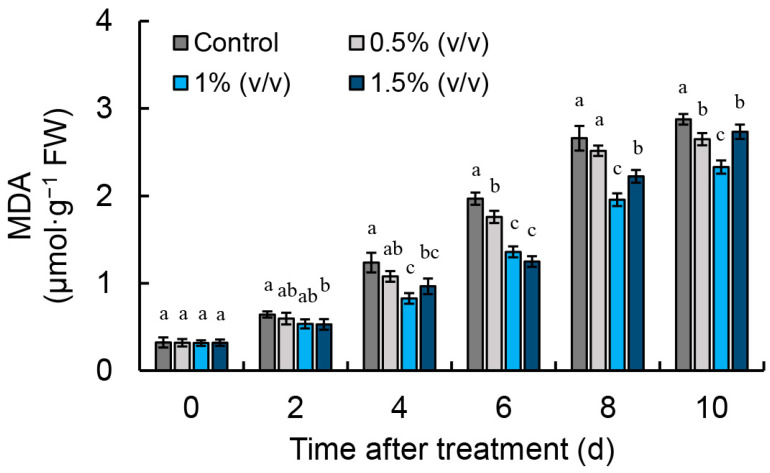
Effect of phytic acid treatment on MDA content in *R. roxburghii* fruit. Different letters indicate statistically significant differences (*p* < 0.05). Vertical bars represent the standard errors of the means (±SE).

From the comprehensive comparison in Figure 1, it can be observed that phytic acid treatment at different concentrations can inhibit the production of MDA in *R. roxburghii* fruit. Compared to other concentrations, the influence of phytic acid at concentration 1% (*v*/*v*) is more significant. For subsequent experiments, a concentration of 1% (*v*/*v*) phytic acid was used.

### 2.2. Effect of Phytic Acid on ROS Metabolism

**Figure 2:**
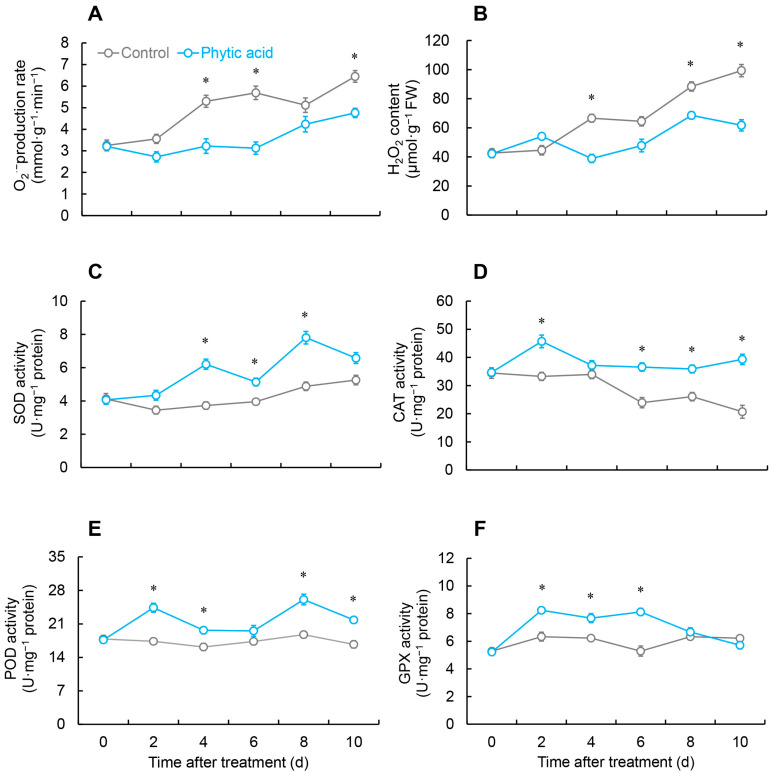
Changes in O_2_^•−^ production rate (**A**), H_2_O_2_ content (**B**), and the activities of SOD (**C**), CAT (**D**), POD (**E**), and GPX (**F**) in *R. roxburghii* fruit after phytic acid treatment during storage at room temperature. * denotes significant difference at the level of *p* < 0.05. Vertical bars represent the standard errors of the means (±SE).

The rate of superoxide anions (O_2_^•−^) production in the control fruit remained consistently elevated compared to the phytic acid-treated group over the entire storage, and on days 4, 6, and 10, it was considerably larger than the treatment group by 1.6, 1.8, and 1.3 times, respectively (Figure 2A).

Phytic acid treatment reduced the H_2_O_2_ content in postharvest *R. roxburghii* fruit. As illustrated in Figure 2B, on days 4, 8, and 10, the H_2_O_2_ content in the treated group was remarkably reduced compared with the control.

The SOD content in the control fruit dropped from days 0 to 2, and then slowly climbed up until day 10. In contrast, the phytic acid-treated group showed two peaks in SOD activity on days 4 and 8, with values remarkably exceeding the control by 1.6 and 1.5 times, respectively (Figure 2C).

After phytic acid treatment, the CAT activity in *R. roxburghii* fruit spiked from days 0 to 2, then saw a reduction from days 2 to 4, followed by a steady level, and was markedly larger than the control on days 2, 6, and 10. The CAT activity in the control group showed little variation from days 0 to 4 and then decreased thereafter (Figure 2D).

The POD activity in the control fruit remained relatively stable over the course of storage, with no remarkable changes. In contrast, the treatment fruit showed significantly higher POD activity on days 2, 4, 8, and 10, with distinct peaks on days 2 and 8, which were 1.4 and 1.3 times compared with the control group (Figure 2E).

The GPX activity in the phytic acid treated fruit rapidly raised from days 0 to 2, remained at a high level from days 2 to 6, appreciably larger than the control, and then decreased (Figure 2F).

### 2.3. Effect of Phytic Acid on Ascorbate–Glutathione Cycle

**Figure 3:**
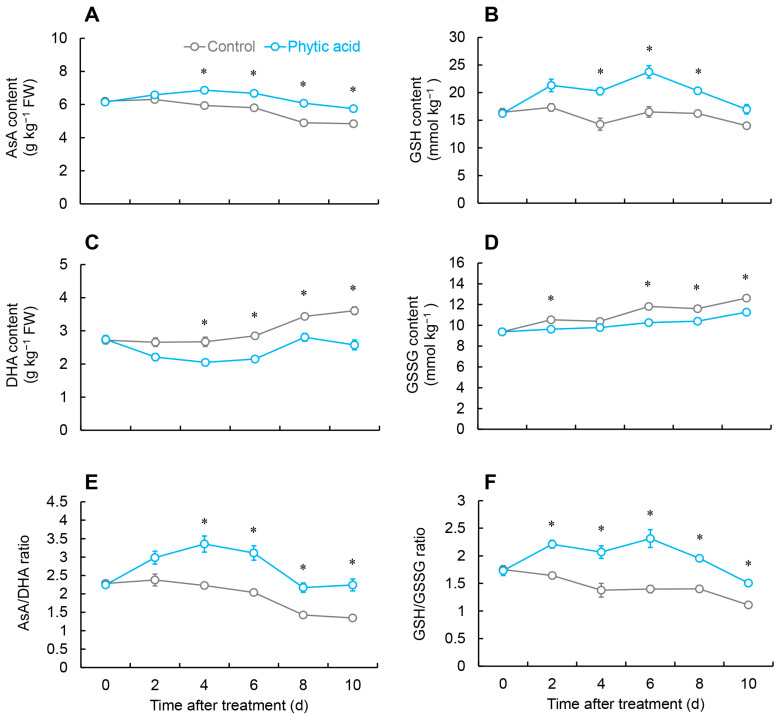
Changes in the AsA (**A**), GSH (**B**), DHA (**C**), and GSSG (**D**) content, and ratios of AsA/DHA (**E**) and GSH/GSSG (**F**) in *R. roxburghii* fruit after phytic acid treatment during storage at room temperature. * denotes significant difference at the level of *p* < 0.05. Vertical bars represent the standard errors of the means (±SE).

As shown in Figure 3A, the AsA content in the control decreased constantly from days 2 to 8, whereas in the phytic acid-treated group, from days 0 to 4, the AsA content showed an increase, and it stayed notably greater than the control throughout days 4 to 10.

The GSH content peaked on day 6 following phytic acid treatment, and it was 1.4 times more than the control. Additionally, the GSH content was consistently significantly higher than the control from days 4 to 8 (Figure 3B).

The control fruit exhibited substantially larger dehydroascorbic acid (DHA) content than the treatment group at every time point, except for day 2 (Figure 3C).

The control and treatment groups experienced a rise in oxidized glutathione (GSSG) content throughout the experimental period. On days 2, 6, and 10, GSSG levels in the phytic acid-treated group were distinctly elevated compared to the untreated group (Figure 3D).

The AsA/DHA ratio in treated fruit increased from days 0 to 4, then fell, and from days 4 to 10, it was noticeably better than that of the control group (Figure 3E).

As shown in Figure 3F, the GSH/GSSG ratio in the control group continuously weakened, while in the phytic acid-treated fruit, it showed an increase from days 0 to 2, followed by fluctuations from days 2 to 6, and then a decrease. Across the entire storage period, the GSH/GSSG ratio in the treated fruit remained substantially elevated compared with the control.

**Figure 4:**
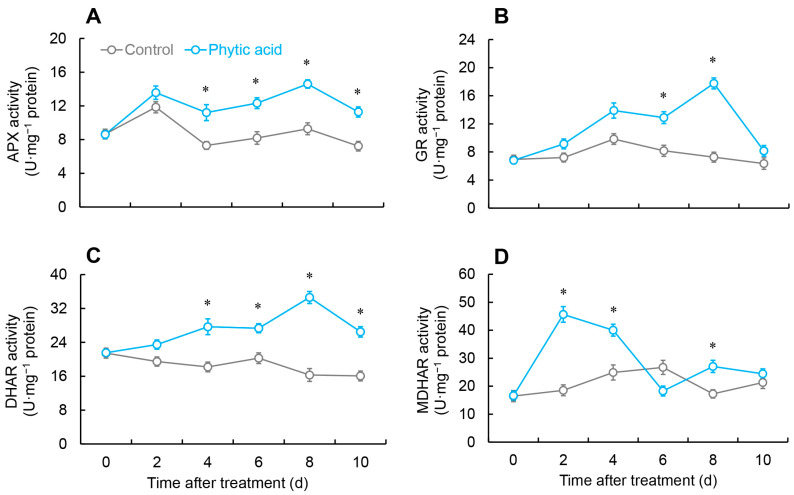
Changes in the activities of APX (**A**), GR (**B**), DHAR (**C**), and MDHAR (**D**) in *R. roxburghii* fruit after phytic acid treatment during storage at room temperature. * denotes significant difference at the level of *p* < 0.05. Vertical bars represent the standard errors of the means (±SE).

The APX activity pattern was similar in the treatment and control groups, rising from days 0 to 2, then declining, followed by a rise again from days 4 to 8. However, the APX activity in the phytic acid-treated fruit was markedly elevated compared to the control from days 4 to 10 (Figure 4A).

The GR activity in *R. roxburghii* fruit peaked on day 8 after phytic acid treatment, being 2.4 times greater than the control, whose peak occurred on day 4 (Figure 4B).

The DHAR activity in the treated fruit followed a similar trend to that of GR, with a peak on day 8 and remarkably improved compared to the control from days 4 to 10 (Figure 4C).

The MDHAR activity in the treated group rapidly increased and peaked during the first 2 days, then declined from days 2 to 6, followed by fluctuations from days 6 to 10. The MDHAR activity was considerably elevated compared with the control on days 2, 4, and 8. Conversely, the MDHAR activity in the control group rose gradually from days 0 to 6, before showing fluctuations (Figure 4D).

### 2.4. Key Enzyme Gene Expressions for ROS and AsA-GSH Metabolism in R. roxburghii Fruit

**Figure 5:**
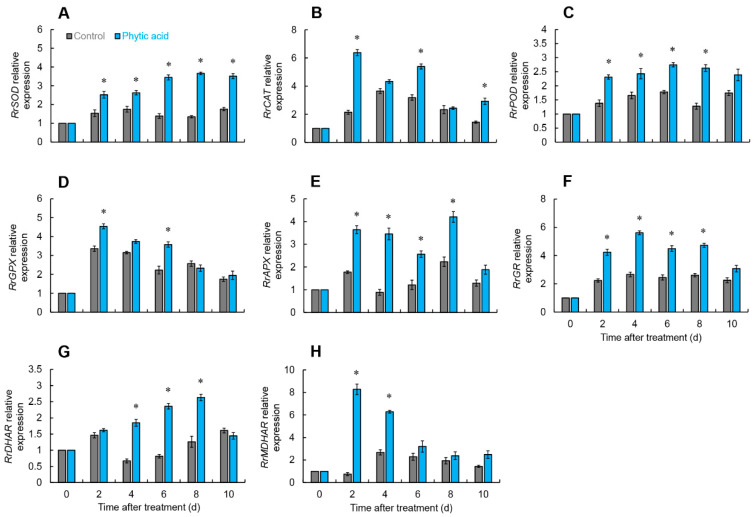
Gene expressions of *RrSOD* (**A**), *RrCAT* (**B**), *RrPOD* (**C**), *RrGPX* (**D**), *RrAPX* (**E**), *RrGR* (**F**), *RrDHAR* (**G**), and *RrMDHAR* (**H**) in *R. roxburghii* fruit after phytic acid treatment during storage at room temperature. * denotes significant difference at the level of *p* < 0.05. Vertical bars represent the standard errors of the means (±SE).

The *RrSOD* expression in the treated group was substantially elevated compared with the control for the duration of storage, peaking on day 8, at 2.7 times the control (Figure 5A).

The *RrCAT* expression in the phytic acid-treated group was considerably greater than the control on days 2, 6, and 10, being 2.9, 1.6, and 2 times higher, respectively (Figure 5B).

The *RrPOD* expression in the treatment group increased from days 0 to 6 and peaked, as seen in Figure 5C. From days 2 to 8, it was clearly larger than in the control group.

The expression of *RrGPX* in the control and treated groups showed comparable changes, rapidly increasing to a peak from days 0 to 2, then gradually decreasing. However, as shown in Figure 5D, on days 2 and 6, the treated group revealed a significant boost in *RrGPX* expression compared with the control.

Phytic acid treatment appreciably raised the *RrAPX* expression in *R. roxburghii* fruit from days 2 to 8 (Figure 5E).

After phytic acid treatment, the *RrGR* expression elevated from days 0 to 4, then dropped, and distinctly greater than the control on days 2 to 8, while the *RrGR* expression in the control rose from days 0 to 2 and then fluctuated (Figure 5F).

The *RrDHAR* expression in the treated group continuously increased from days 0 to 8, then decreased, and was remarkably larger than the control group from days 4 to 8 (Figure 5G).

The *RrMDHAR* expression in the treated group showed a marked increase from days 2 to 4, being 11 and 2.3 times larger than the control on these days. At other time points, the treated and control groups exhibited similar results with no significant difference (Figure 5H).

## 3. Discussion

During the aging process, the balance of ROS metabolism in plants weakens, resulting in a rise in free radical content and a decrease in the ability to scavenge these radicals. When ROS levels are too high, they can lead to damage of proteins, lipids, and DNA, which subsequently results in membrane lipid oxidation, biological membrane degradation, and metabolic disorders [20,21,22]. Antioxidant enzymes in plants help eliminate excess ROS, maintaining the balance of ROS [23]. Within cells, SOD is a key source of H_2_O_2_, converting O_2_^•−^ into the more stable H_2_O_2_, thus mitigating oxidative stress in plants. Subsequently, GPX and CAT decompose H_2_O_2_ into H_2_O and O_2_. In addition, POD can work in conjunction with CAT and SOD to remove excess free radicals [24,25]. This study demonstrated that phytic acid treatment can significantly inhibit the increase in O_2_^•−^ production rate and H_2_O_2_ content during the storage of *R. roxburghii* fruit (Figure 2A,B) while also suppressing the production of MDA (Figure 1). Previous research by Wang et al. confirmed that phytic acid can directly neutralize scavenge free radicals and inhibit lipid peroxidation, which is consistent with our findings [17]. The combination of 1-methylcyclopropene and phytic acid treatment impactfully prevents the MDA content in fresh-cut peaches, helping to maintain postharvest quality [19]. This research demonstrated that phytic acid treatment caused a notable boost in the activities and expressions of SOD, CAT, and POD in the fruit of *R. roxburghii* (Figure 2C–E and Figure 5A–C). These findings suggested that postharvest phytic acid treatment can activate the activity of antioxidant enzymes, which in turn helps regulate ROS levels and protects the fruit from oxidative damage. Treatment with phytic acid on fresh-cut apples led to a substantial rise in the activities of SOD and CAT, while the contents of H_2_O_2_ and MDA were effectively suppressed [18]. Previous research by Foku et al. found that the application of phytic acid in grape berries resulted in a significant reduction in H_2_O_2_, O_2_^•−^, and MDA levels, in addition to a rise in the activity of SOD, CAT, and POD [5]. Analogous results were also observed in apples following phytic acid treatment [26]. Based on the above findings, phytic acid treatment appears to activate the gene expression and activity of key antioxidant enzymes, including SOD, CAT, and POD, thereby reducing excessive ROS in *R. roxburghii* fruit, ensuring membrane integrity, and slowing down fruit senescence.

The AsA-GSH cycle plays a crucial role in the clearance and balance of ROS in fruits and vegetables. Many previous studies confirmed that by scavenging surplus ROS, the AsA-GSH cycle strengthens plant resistance and slows down postharvest senescence [27,28]. In the AsA-GSH cycle, APX is responsible for converting H_2_O_2_ into H_2_O and O_2_, with AsA acting as the electron donor. Through this reaction, AsA is oxidized to form Monodehydroascorbic acid (MDHA) and DHA [29]. Additionally, by serving as an electron donor, DHAR and GSH can help decrease DHA back to AsA [30]. MDHAR can be used to convert MDHA back to AsA. According to this study, phytic acid treatment heightened both the activities and expressions of APX, DHAR, and MDHAR (Figure 4A,C,D and Figure 5E,G,H) while effectively increasing AsA content and reducing DHA levels (Figure 3A,C). A study by Foku et al. found that phytic acid treatment elevated the activity and gene expression of DHAR, APX, MDHAR and GR in grapefruit [5]. As a key non-enzymatic antioxidant in the AsA-GSH cycle, GSH is maintained in balance by GR, which converts GSSG back to GSH [31]. The GPX cycle is another important enzymatic mechanism for ROS scavenging in plants and fruits. GPX, which reduces H_2_O_2_ using GSH as an electron donor, producing GSSG, and GR, which facilitates the regeneration of GSH using NADPH as the electron donor, consequently reducing ROS [32]. Furthermore, the AsA/DHA and GSH/GSSG ratios are also crucial indicators of the cellular redox status, which indicates the functionality of the AsA-GSH cycle [29]. This research revealed that phytic acid treatment could enhance the activity and gene expression of GR and GPX (Figure 2F, Figure 4B and Figure 5D,F) and increase GSH content while reducing GSSG levels (Figure 3B,D). Additionally, the control group of *R. roxburghii* fruit showed a lower AsA/DHA and GSH/GSSG ratio, indicating a stronger overall oxidative state. In contrast, after phytic acid treatment, the AsA/DHA and GSH/GSSG ratios were greater, indicating a stronger reduced state within the fruit and enhanced antioxidant capacity (Figure 3E,F). The results suggested that phytic acid treatment can enhance the AsA-GSH cycle in *R. roxburghii* fruits, maintain ROS balance, and thus ensure that the postharvest *R. roxburghii* fruits remain in a more reduced state. As a new postharvest preservation method, phytic acid merits further thorough investigation. Future research might concentrate on clarifying the precise methods by which related transcription factors direct the regulation of genes linked to AsA metabolism, as well as how phytic acid controls the expression of these genes. We also looked more closely at the possible industrial uses of this treatment, such as the creation of integrated preservation technologies that provide scalable solutions for commercial fruit preservation on a large scale by combining phytic acid with physical storage techniques like low-temperature storage, controlled atmosphere storage, or modified atmosphere packaging.

## 4. Materials and Methods

### 4.1. R. roxburghii, Chemicals and Treatment

The fruits of *R. roxburghii* were harvested from an orchard located in Longli County, Guizhou Province, China, with no defects or damage. Following collection, the *G. elata* was brought to the laboratory on the same day, where intact samples without insect damage or mechanical harm were meticulously chosen. Solarbio (Beijing, China) provided support for phytic acid.

The undamaged fruits were washed once with distilled water, then immersed in phytic acid solutions of 0.5%, 1%, and 1.5% (*v*/*v*) for 10 min, using distilled water as the control; the fruits were air-dried at room temperature following the treatment, placed in polyethylene trays, and stored in a climate-controlled chamber at 85% relative humidity and 22 ± 1 °C. Each treatment involved three replicates, with 120 fruits per replicate.

### 4.2. Sample Collection

Tissue samples, taken from 3 to 6 mm below the peel and around the equatorial zone of the fruit, were collected at 0, 2, 4, 6, 8, and 10 days following phytic acid treatment. After being rapidly frozen in liquid nitrogen, the samples were kept at −80 °C for preservation.

### 4.3. Determination of MDA Content

Referring to the method of Ren et al. [33] with modifications, 1.0 g of fruit tissue was taken and homogenized with 5 mL of 100 g/L trichloroacetic acid (TCA) solution. The homogenate was then centrifuged at 12,000× *g* for 20 min at 4 °C. For the blank control, 2 mL of 100 g/L TCA solution was used instead of the extract, and 2 mL of supernatant was collected from the sample. Then, 2 mL of 0.6% thiobarbituric acid (TBA) solution (prepared with TCA) was added. After mixing, the solution was boiled for 20 min, cooled, and centrifuged again. Following measurements of absorbance at 450, 532, and 600 nm, the MDA content was calculated and reported as μmol g^−1^ fresh weight (FW).

### 4.4. Determination of O_2_^•−^ Production Rate, H_2_O_2_ Content, and ROS Metabolism-Related Enzyme Activity

Then, 5 mL of 100 mmol/L phosphate buffer (pH 7.8, including 0.1% PVPP (*w*/*v*)) was mixed with 1.0 g of tissue, homogenized on ice, and centrifuged at 9000× *g* for 10 min at 4 °C. The assay was conducted according to the protocol described by Ren et al. [33]. The O_2_^•−^ production rate was expressed as mmol g^−1^ min^−1^.

Referring to the method of Wei et al. [34] with modifications, 1.0 g of fruit tissue was homogenized with 3 mL of cold acetone under ice bath conditions. The homogenate was then centrifuged at 12,000× *g* for 20 min at 4 °C. Moreover, 200 µL of concentrated ammonia and 100 µL of a 20% titanium tetrachloride solution (dissolved in concentrated hydrochloric acid, *v*/*v*) were then combined with 1 mL of the supernatant. After mixing, the reaction was allowed to proceed for 5 min before centrifugation for 15 min. To weaken the pigment interference, cold acetone was used four times to wash the resultant precipitate. Ultimately, 1.5 mL of a 1 mmol/L H_2_SO_4_ solution was used to dissolve the precipitate. The H_2_O_2_ content was measured by absorbance at 410 nm and expressed as μmol g^−1^ FW.

Then, 3.0 mL of 0.05 M phosphate buffer (pH 7.4) enhanced with 5.0 mM dithiothreitol (DTT) and 10 g/L polyvinyl polypyrrolidone (PVPP) comprised the extraction buffer for SOD and CAT. SOD and CAT activities were measured using the Micro Reduced SOD Assay Kit and CAT Assay Kit (Solarbio Life Science, Beijing, China), following the manufacturer’s protocols. POD was extracted using 0.05 M phosphate buffer (pH 7.5), containing 1 mL/L Triton X-100 and 10 g/L polyvinyl polypyrrolidone (PVPP). POD activity was determined following the method of Ding et al. [35] by monitoring the oxidation of guaiacol to tetraguaiacol spectrophotometrically at 470 nm over 2 min. Enzyme activity was expressed as U mg^−1^ protein, where 1 U corresponds to 0.01 Δ470 min^−1^. The GPX activity was detected using an assay kit (AKPR014, Boxbio, Beijing, China). One unit of activity is defined as a decrease of 1 μM GSH per minute at 37 °C.

### 4.5. Determination of GSH, AsA, GSSG, DHA Content, and AsA-GSH Metabolism-Related Enzyme Activity

GSH and AsA contents were measured using a commercially available assay kit (A009-1-1 and A006-1-1, Nanjing Jiancheng Bioengineering, Nanjing, China) and reported in mmol kg^−1^ FW and g kg^−1^ FW, respectively. An assay kit (AKVI008 and AKPR009, Boxbio, Beijing, China) was used to measure the levels of DHA and GSSG. The results were then represented in the appropriate units of g kg^−1^ FW and mmol kg^−1^ FW, respectively.

Ascorbate peroxidase (APX), glutathione reductase (GR), monodehydroascorbate reductase (MDHAR), and dehydroascorbate reductase (DHAR) activities were determined following the method of Ding et al. [35]. Enzyme extraction was performed using specific phosphate buffers: 0.1 M (pH 7.5) with 1 mM EDTA for APX, DHAR, and GR, and 0.04 M (pH 7.5) supplemented with 20 g/L PVPP and 5 mM β-mercaptoethanol for MDHAR. APX activity was assayed based on the oxidation of ascorbate at 340 nm, while GR activity was determined by monitoring NADPH oxidation at the same wavelength. MDHAR and DHAR activities were measured at 340 nm and 290 nm, respectively. Enzyme activities were expressed as U mg^−1^ protein, where 1 U corresponded to 0.01 ΔA340 min^−1^ for APX, GR, and MDHAR, and 0.01 ΔA290 min^−1^ for DHAR.

### 4.6. RNA Extraction and First-Strand cDNA Synthesis

Following the manufacturer’s instructions, 1.0 g of *R. roxburghii* was subjected to RNA isolation using the RNeasy Plant Mini Kit (Takara, Shiga Japan). Following all instructions exactly, 1 µg of total RNA was processed using the Reverse-iT™ 1st Strand Synthesis Kit (Takara, Japan) for cDNA synthesis.

### 4.7. Real-Time Quantitative (RT-PCR)

The RT-PCR reaction mix consisted of 1 µL of cDNA template, 1 µL of 10 µM forward and reverse primers, and 10 µL of SYBR™ Green qPCR Master Mix, giving a total volume of 20 µL per well. Analysis was conducted using the ABI PRISM™ 7000 Sequence Detection System (Applied Biosystems, Foster City, California, USA), with Actin as the reference gene. Samples were analyzed in triplicate, and the primers used are provided in Table S1. Relative gene expression was determined using the 2^−ΔΔCt^ method, normalizing to Actin.

### 4.8. Data Analysis

Version 19.0 of the SPSS software was used to analyze the data. Applying a one-way analysis of variance (ANOVA), significant differences were identified at *p* < 0.05. A Student’s *t*-test was used for pairwise comparisons between the phytic acid treatment and the control.

## 5. Conclusions

**Figure 6:**
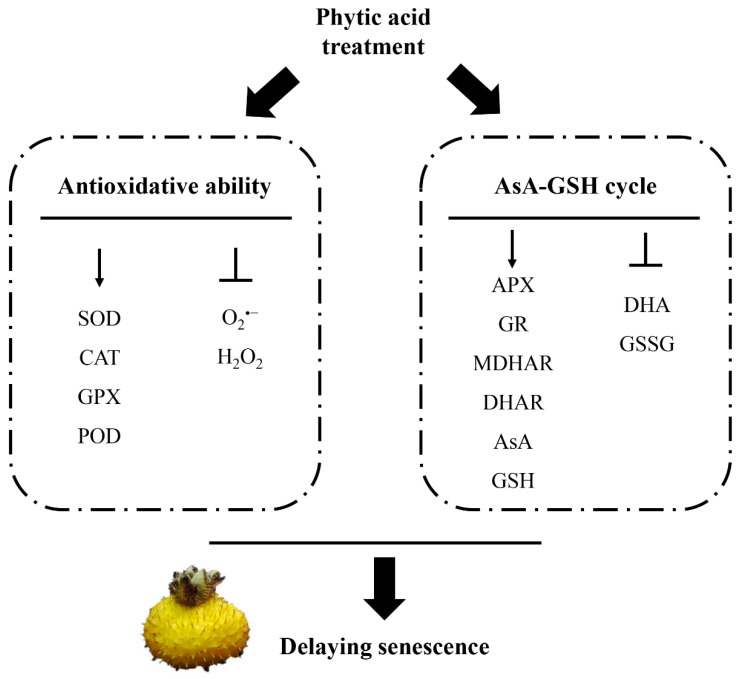
A speculation mechanism to explain the role of phytic acid in delaying senescence in *R. roxburghii* fruit.

In summary, the findings of this study indicated that phytic acid treatment can elevate the activity of postharvest antioxidant-related enzymes in *R. roxburghii* fruit, reduce oxidative damage to cells, and inhibit MDA production. Phytic acid treatment also activates the AsA-GSH cycle, boosting the activity of key enzymes such as APX, GR, DHAR, and MDHAR. Additionally, it increases the content of non-enzymatic antioxidants like AsA and GSH, maintaining the fruit in a more reduced state, which effectively preserves the postharvest quality of zymes in *R. roxburghii* fruit. A speculative mechanism for how phytic acid delays the senescence of *R. roxburghii* fruit is shown in Figure 6, along with suggestions for future, more in-depth investigation.

## Data Availability

The original contributions presented in the study are included in the article; further inquiries can be directed to the corresponding authors.

## Supplementary Materials

The following supporting information can be downloaded at: https://www.mdpi.com/article/10.3390/ijms26010098/s1.
