# Supplementary material for: Phytic Acid Delays the Senescence of Rosa roxburghii Fruit by Regulating Antioxidant Capacity and the Ascorbate–Glutathione Cycle
Source: Int J Mol Sci. 2024 Dec 26;26(1):98. doi: 10.3390/ijms26010098 (PMC11720362; doi:10.3390/ijms26010098)
Supplement: Supplementary file 1 [file ijms-26-00098-s001.zip › ijms-3379543-supplementary.pdf]

Supplementary material

Table S1 Primer sequences used for real-time PCR.

GENE	PRIMER SEQUENCE	GENBANK ID
RrSOD	Forward: ACCACCAGAAGCACCACCAG	Rro07G042730
	Reverse: GTTGATATGACCTCCGCCGTTG	
RrCAT	Forward: AGTTGGCCGTTTGGTTCTGA	Rro05G031190
	Reverse: AGGGACAATAATGGCAGGGC	
RrPOD	Forward: AAGAAGCCTCGGACAACATTATGC	Rro02G021710
	Reverse: CAATGGAGTGTGACCCTGAAAGC	
RrGPX	Forward: TGGCAACCCAACCTACTGAG	Rro05G018480
	Reverse: CCACATCATGCCCCTTAGCA	
RrAPX	Forward: GGGTTCTGACCACTTGAGGG	KC782562
	Reverse: AGGTGTGACCACCAGAGAGA	
RrGR	Forward: ATCCGGACGGCAAGAAAAGT	Rro04G000730
	Reverse: GAATCTGGCCCACACATGGA	
RrDHAR	Forward: GTTCGGAACAGGCTTTGCTT	KC782570
	Reverse: GACCTTCTCCCCAGCAATGT	
RrMDHAR	Forward: GCAGATTTGCGTGCATGGTC	GU552461
	Reverse: ATGGAGTGCCTTTCGTCTGG	
Rractin	Forward: TGCCATCCTTCGTCTTGACC	GU552463
	Reverse: CGCTCTGCAGTGGTAGTGAA	
